# Developing a novel tool to assess the ability to self-administer medication – A systematic evaluation of patients’ video recordings in the ABLYMED study

**DOI:** 10.3389/fmed.2023.1040528

**Published:** 2023-02-16

**Authors:** Anneke Luegering, Robert Langner, Stefan Wilm, Thorsten R. Doeppner, Dirk M. Hermann, Helmut Frohnhofen, Janine Gronewold

**Affiliations:** ^1^Hospital Pharmacy, University Hospital Düsseldorf, Düsseldorf, Germany; ^2^Institute of Systems Neuroscience, University Hospital Düsseldorf, Düsseldorf, Germany; ^3^Institute of Neuroscience and Medicine (INM-7: Brain and Behaviour), Research Centre Jülich, Jülich, Germany; ^4^Institute of General Practice, Centre for Health and Society (chs), University Hospital Düsseldorf, Düsseldorf, Germany; ^5^Department of Neurology, University Hospital Giessen, Giessen, Germany; ^6^Research Institute for Health Sciences and Technologies (SABITA), Istanbul Medipol University, Istanbul, Türkiye; ^7^Department of Anatomy and Cell Biology, Medical University of Varna, Varna, Bulgaria; ^8^Department of Neurology, University of Göttingen, Göttingen, Germany; ^9^Department of Neurology and Center for Translational Neuro- and Behavioral Sciences (C-TNBS), University Hospital Essen, University Duisburg-Essen, Essen, Germany; ^10^Department of Orthopedics and Trauma Surgery, University Hospital Düsseldorf, Düsseldorf, Germany; ^11^Department of Medicine, Geriatrics, Faculty of Health, University Witten-Herdecke, Witten, Germany

**Keywords:** self administration, aged, medication management problems, self-reported ability, video recordings, rating procedure

## Abstract

**Background:**

Older people often experience medication management problems due to multimorbidity, polypharmacy and medication complexity. There is often a large gap between patients’ self-reported and actual abilities to handle the self-administration of their medication. Here we report on the development and evaluation of a new tool to assess the ability of non-demented hospitalized patients to self-administer medication in different dosage forms. To this end, we video-recorded the patients’ medication management performance and implemented a novel assessment scheme, which was applied by several independent raters.

**Methods:**

Sixty-seven in-patients ≥70 years of age and regularly taking ≥5 different drugs autonomously of the ABLYMED study agreed to the video recording of their medication management performance with five different dosage forms. All raters underwent a training and applied a standardized assessment form and written guide with rating rules for evaluation. In a pilot phase, video recordings of three patients were rated by 19 raters (15 medical students, two expert raters to determine a reference standard, and two main raters who later rated the total sample). In the rating phase, based on the ratings obtained from the two main raters, we determined interrater (assessed every section of 20 patients as agreement between the raters at one point of time) and intrarater (assessed as consistency within each rater across three points of time) agreement by intraclass correlation analysis.

**Results:**

In the pilot phase we obtained an overall sufficient agreement pattern, with an adjustment of the rating rules for patches. In the rating phase we achieved satisfactory agreement between the two raters (interrater reliability) and across different points of time (intrarater reliability). For two dosage forms (eye-drops and pen), rater training needed to be repeated to reach satisfactory levels.

**Discussion:**

Our novel rating procedure was found to be objective, valid and reproducible, given appropriate training of the raters. Our findings are an important part of a larger research project to implement a novel assessment for the ability to self-administer medication in different dosage forms. Further, they can support the development of patient trainings to improve medication management and secure independent living.

**Clinical Trial Registration:** DRKS00025788, date of registration: 07/09/2021.

## Introduction

Adequate medication management is essential for a successful pharmacotherapy (1). Especially in older patients, impairments in physical and mental functions and increasing medication complexity make medication management challenging. This can lead to lower adherence, more medication errors and finally suboptimal treatment outcomes (1–3). Despite the high prevalence of problems with medication management in older people, these problems often remain unrecognized (4). With increasing age, comorbidity increases, and medication regimens become more complex, leading to higher workload in patients with mostly reduced capacity (5, 6). Medication regimen complexity is driven by the number of medications in different dosage forms, frequency of intake and required manipulations of medications. Examples for complex medication procedures are opening medication packages when sealed, releasing pills from a blister, cutting pills and opening child resistant bottles (4).

Therefore, in older adults, daily medication preparation is often time consuming, associated with coping strategies that differ from the instructions by the manufacturer, or with the inability to handle medication at all. Handling errors in daily medication preparation increase the risk of nonadherence (4, 7). Of note, such impairments are often not realized by patients and thus they do not report medication management problems: there is often a large gap between patients’ self-reported and actual medication management skills (4).

Unfortunately, previous studies assessing medication management performance in older patients are scarce (8). To the best of our knowledge, most previous studies describe handling errors only of one selected dosage form such as inhalers, eye drops or pills. In addition, there is no instrument recommended for use in clinical practice which objectively measures medication self-management capacity (9). Furthermore, most findings were based on single person judgements or patients’ self-reports (10–13). However, such methods bear the risk of bias. Nevertheless, eye-drop instillation has already been examined by a masked analysis of video recordings of patients self-instilling eye drops covering the aspects efficiency, safety and efficacy of eye-drop instillation, assessed by three raters (14).

For a comprehensive analysis of medication management, all common dosage forms should be evaluated, and in addition to patient’s self-report, an objective, reliable, and ecologically valid quantitative assessment of performance based on behavioral observations in standardized test settings should be applied, involving multiple raters at multiple points in time.

The ABLYMED (8) study aims at developing a new tool to assess the ability to self-administer medication in non-demented hospitalized patients from the University Hospital Düsseldorf. The evaluation involves both subjective self-report measures and performance assessments provided by at least two raters. We used original packaging of medication that cover most medication formulations to create ecologically valid test scenarios.

One part of the ABLYMED study is a video-based evaluation of the self-administration of medication in different dosage forms. We made video recordings of each patient performing different tasks of medication management. Each study patient self-administered five different placebo dosage forms of medication in a video-instructed way. Each patient’s self-administration performances, as captured by the video recordings, was than rated via systematic assessment procedure. Here we describe the development of the evaluation procedure used to reliably assess video recorded medication management performance in the ABLYMED study.

Since we live in an aging society and in 2030 seventy-one million people are expected to be over 65 years old, treatment of senior patients is getting more important, independently from the medical subject (15). Besides, physicians often overestimate their patients’ cognitive and motor abilities needed for adequate self-administration of medication (16). Thus, our research is of high clinical and practical relevance.

## Materials and methods

### Setting and video evaluation procedure

The ABLYMED study recruited 100 non-demented patients from the University Hospital Duesseldorf ≥70 years of age regularly taking ≥5 different drugs autonomously. The median age was 79 years (74;84) and 50% were female. Of these patients, *n* = 67 agreed to the video recording of their medication management performance with placebo drugs. The study was approved by the ethics committee at the medical faculty of the Heinrich Heine University Düsseldorf (reference number 2021-1435). All patients gave written informed consent before entering the study.

The video recordings were made by AL (pharmacist) in the patient’s rooms with a smart phone camera. To minimize intrusiveness, no further equipment was used. The video recordings showed patient’s hands and arms, but not their faces and the videos are muted, for privacy protection. Patients performed the self-administration tasks in a sitting position. If patients were unable to perform steps of administration tasks, they received support by AL: verbal support could be given twice for each step of medication administration, if this did not help to complete the step, the video was interrupted, and practical assistance was given. Afterwards, the video recording started again, and the patient continued with the next step. If practical assistance was necessary, the rater evaluated this step of administration with “not possible” as described in the rating rules (see Supplementary Table S2). If there was no following video, all further administration steps were also not possible. In this way, all steps of medication administration could be evaluated comprehensively. Verbal support was recognizable for the raters by ALs gestures on the video.

For each patient, five videos were recorded (five dosage forms [tablets, eye-drops, oral drops, insulin pen and patches]). Videos were evaluated by up to 19 raters using a standardized assessment form. The assessment form was developed by the two experts HF (internist, geriatrician) and AL based on viewing a few video recordings, literature review and following expert discussion including a statistician to reach consent. The quality of the assessment form was assessed by inter- and intrarater agreement. Interrater agreement was determined as the degree of agreement between different raters at one point of time. Intrarater agreement was determined as the degree of agreement (per rater) across three different points of time.

All raters got a training containing explanations to the instructional videos (see Supplementary Videos S1–S6), which were used to instruct the patients, and to the standardized assessment form (see Supplementary Table S1). Furthermore, the raters received a written guide with rating rules (see Supplementary Table S2). Thereafter, they evaluated the video recordings of the patients’ medication management performance.

The assessment form contained a 5-point Likert scale for each step of the medication administration (5 = not possible, meaning practical assistance needed or interruption; 4 = severe difficulties, meaning execution hardly possible or success of therapy at risk; 3 = moderate difficulties, meaning execution significantly slowed down; 2 = mild difficulties, meaning execution slightly slowed down; 1 = no difficulties, meaning correct and fluid execution). In some cases, the assessment form contained the choice between correct or incorrect (1 = correct and 2 = incorrect).

The video raters were 15 random medical students (R1 - R15), JG (psychologist and epidemiologist), TD (neurologist), AL, and HF. The students voted anonymously during a lecture. JG and TD got access to the video recordings via a password-protected cloud solution and independently sent their evaluation results on the standardized assessment form via email. AL and HF evaluated the videos together and discussed to reach a consensus to set the reference standard.

### Statistical analysis

For each dosage form, we summed up the scores for each administration step for each rater, leading to a continuous sum score, and analyzed the interrater agreement by intraclass correlation coefficient (ICC). For analyzing intrarater agreement, we analyzed agreements per rater between the sum score of the first evaluation, a second evaluation repeated after 2 weeks and a third evaluation after 4 weeks of one patient, using ICC as well (17). Missing data were imputed by the median (applies only to the evaluation of R1-R15). The dosage form-specific interrater agreement and intrarater agreements are presented as ICC with 95% confidence interval, based on consistency, 2-way mixed-effects model. Data were analyzed using SPSS 22 for Windows (IBM Corporation, Armonk, NY, United States).

### Phases of the video evaluation procedure

The evaluation procedure was divided into two phases: a pilot phase and a rating phase.

#### Pilot phase

First, AL and HF selected videos of three patients that broadly covered the spectrum of no, moderate, and severe handling problems of each dosage form. These videos can be viewed in the supplementary (Supplementary Videos S7–S23). Each patient had five video sequences that showed the self-administration tasks of tablets, eye-drops, oral drops, a pen and a patch.

These videos were first rated by AL and HF who reached a consensus to set the reference standard. Next, 15 randomly selected medical students got a training containing explanations to the instructional videos, the standardized assessment form, and the written guide with rating rules. Thereafter, they rated patients´ medication management performance independently and blinded to the reference standard. Finally, JG and TD got the same training and evaluated the videos independently from each other and blinded to the former results.

**Figure 1:**
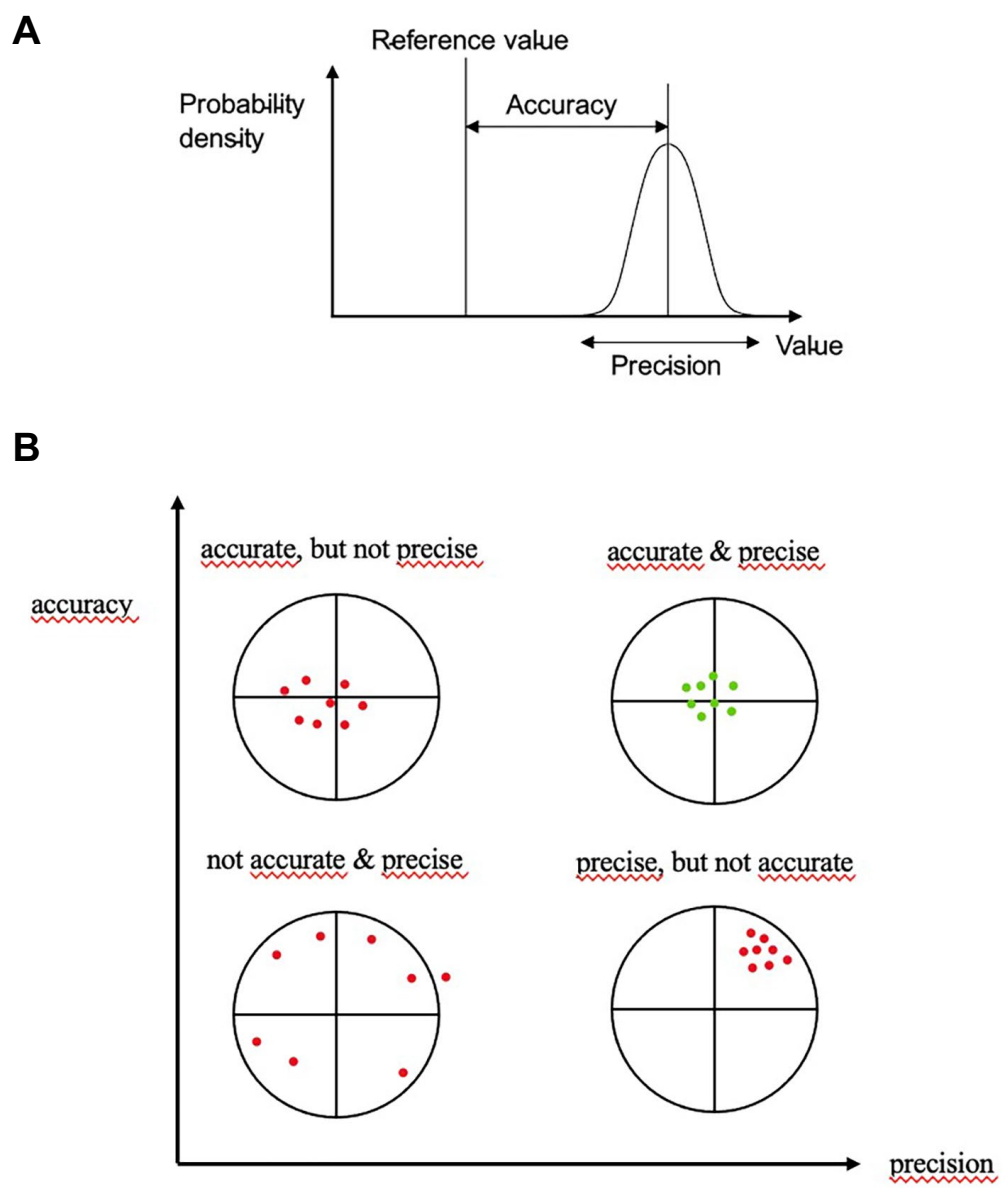
Accuracy is the proximity of assessments to the true value; precision is the degree to which repeated assessments under unchanged conditions show the same results. An ideal assessment should be accurate and precise (upper part Pekaje, creative common license wikimedia, https://commons.wikimedia.org/wiki/File:Accuracy_and_precision.svg, downloaded 25th July, 2022).

To analyze the accuracy and precision of our standardized assessment form, we measured the agreements shown in Table 1 regarding the sum scores for each dosage form. Agreement with the reference standard was used to evaluate accuracy, that is how close a measurement is to the true or accepted value. Interrater agreement was used to evaluate precision, that is how close measurements of different raters of the performance of the same patients are to each other. A measurement system is considered valid if it is both accurate and precise, that is when measurements are all close to and tightly clustered around the true or accepted value (Figure 1).

**Table 1:** Measures of accuracy and precision in the pilot phase.

	Reference standard	Median R1-R15	Rater JG	Rater TD
Reference standard				
Median R1-R15	Accuracy			
Rater JG	Accuracy	Precision		
Rater TD	Accuracy	Precision	Precision	

In case of both satisfactory agreement of student raters as well as JG and TD with the reference standard (consensus of AL and HG), indicating accuracy, and agreement between the raters, indicating precision, the main evaluation phase was started. Satisfactory agreement was defined by ICC ≥0.5, representing at least moderate agreement (18). Otherwise, the assessment form was adapted, and the evaluation repeated until the agreement was satisfactory.

#### Rating phase

JG and TD rated all patient videos independently and blinded to the results of each other in sections of 20 patients. After each section, the interrater reliability was determined. If the agreement (JG vs. TD) was not satisfactory, the training was repeated, and the evaluation repeated until the agreement was satisfactory. We planned this procedure to keep the quality of the ratings consistently high. Besides, AL and HF determined a reference standard for one patient in each section by consensus. At the end of the rating of all patients videos, the agreement between JG and the reference standard and TD and the reference standard was assessed as an additional accuracy check.

Furthermore, JG and TD rated the video recordings of one patient in three points in time (t0, t1 after 2 weeks and t3 after 4 weeks) blinded to the previous ratings to show intrarater consistency over time.

## Results

### Pilot phase

The rating results of the pilot phase are presented in Table 2. For R1-R15, 14 of 225 sum scores were imputed due to missing data. The video recordings of the three selected patients yielded a dosage form-specific satisfactory rater agreement for tablets, eye-drops, oral drops, and insulin pens. The patches showed satisfactory agreement except for three cases (rater JG vs. reference standard, median of R1-R15 vs. rater TD and rater JG vs. rater TD). Therefore, we added the rating rules for patch type two concerning the peeling off of the protective liner (Supplementary Table S2). In summary, we regarded the overall agreement pattern as sufficient to start the main rating phase.

**Table 2:** Dosage form-specific interrater agreement in the pilot phase: ICC with 95% confidence intervals.

	Tablets	Eye-drops	Oral drops	Insulin pen	Patch
Median of R1-R15 versus reference standard	0.97 [−0.14–1.00]	1.00 [1.00–1.00]	0.99 [0.62–1.00]	1.00 [1.00–1.00]	0.57 [−15.71–0.99]
Rater JG versus reference standard	0.90 [−3.08–1.00]	0.92 [−2.00–1.00]	0.96 [−0.56–1.00]	0.99 [−2.83–1.00]	−2.00 [−116.00–0.92]
Rater TD versus reference standard	0.79 [−7.21–1.00]	0.89 [−3.33–1.00]	0.96 [−0.56–1.00]	0.96 [−24.91–1.00]	0.67 [−12.00–0.99]
R1-R15	0.99 [0.96–1.00]	0.99 [0.94–1.00]	1.00 [0.98–1.00]	0.98 [0.93–1.00]	0.70 [−0.28–0.99]
Median of R1-R15 versus rater JG	0.75 [−8.75–0.99]	0.92 [−2.00–1.00]	0.98 [0.03–1.00]	0.99 [0.72–1.00]	0.57 [−15.71–0.99]
Median of R1-R15 versus rater TD	0.54 [−17.05–0.99]	0.89 [−3.33–1.00]	0.98 [0.03–1.00]	0.96 [−0.44–1.00]	0.00 [−38.00–0.97]
Rater JG versus rater TD	0.95 [−0.86–1.00]	0.77 [−7.81–0.99]	1.00 [1.00–1.00]	0.98 [0.25–1.00]	0.00 [−38.00–0.97]

### Rating phase

Within two months, JG and TD rated all videos of 67 patients. The rating results for each section are presented in Table 3.

**Table 3:** Dosage form-specific rating results: ICC with 95% confidence intervals.

Dosage form-specific interrater agreement for patient 1–20					
	Tablets	Eye-drops	Oral drops	Insulin pen	Patch
Rater JG versus TD	0.83 [0.54–0.94]	−0.09 [−2.02–0.60]	0.75 [0.32–0.91]	0.89 [0.72–0.96]	0.83 [0.56–0.94]
**Dosage form-specific interrater agreement for patient 1–20 after second evaluation of eye-drops**					
	**Tablets**	**Eye-drops**	**Oral drops**	**Insulin pen**	**Patch**
Rater JG versus TD	0.83 [0.54–0.94]	0.79 [0.43–0.93]	0.75 [0.32–0.91]	0.89 [0.72–0.96]	0.83 [0.56–0.94]
**Dosage form-specific interrater agreement for patient 21–40**					
	**Tablets**	**Eye-drops**	**Oral drops**	**Insulin pen**	**Patch**
Rater JG versus TD	0.67 [−0.35–0.92]	0.95 [0.79–0.99]	0.89 [0.49–0.97]	0.28 [−2.58–0.86]	0.99 [0.95–1.00]
**Dosage form-specific interrater agreement for patient 21–40 after second evaluation of the pen**					
	**Tablets**	**Eye-drops**	**Oral drops**	**Insulin pen**	**Patch**
Rater JG versus TD	0.67 [−0.35–0.92]	0.95 [0.79–0.99]	0.89 [0.49–0.97]	0.87 [0.34–0.97]	0.99 [0.95–1.00]
**Dosage form-specific interrater agreement for patient 41–60**					
	**Tablets**	**Eye-drops**	**Oral drops**	**Insulin pen**	**Patch**
Rater JG versus TD	0.90 [0.68–0.97]	0.92 [0.73–0.98]	0.91 [0.71–0.97]	0.82 [0.28–0.96]	0.98 [0.93–0.99]
**Dosage form-specific interrater agreement for patient 61–80**					
	**Tablets**	**Eye-drops**	**Oral drops**	**Insulin pen**	**Patch**
Rater JG versus TD	0.97 [0.91–0.99]	0.87 [0.59–0.96]	0.78 [0.28–0.93]	0.97 [0.89–0.99]	0.97 [0.91–0.99]
**Dosage form-specific interrater agreement for patient 81–100**					
	**Tablets**	**Eye-drops**	**Oral drops**	**Insulin pen**	**Patch**
Rater JG versus TD	0.73 [−0.10–0.93]	0.76 [0.10–0.94]	0.99 [0.95–1.00]	0.98 [0.93–1.00]	0.98 [0.91–0.99]

In the first section (patient-ID 1–20) there was satisfactory interrater agreement for tablets, oral drops, insulin pens and patches. For eye-drops, poor agreement was observed. Therefore, the rating rules for eye-drops application performance were adapted (evaluation was based on the number of grasping movements: one to two: no difficulties, three to four: mild difficulties, five to six: moderate difficulties, more than six: severe difficulties) (see Supplementary Table S2) and training for the dosage form eye-drops was repeated. Afterwards, JG and TD re-evaluated the eye-drops in this section again taking the new rating rules into account and achieved satisfactory interrater agreement.

In the second section (patient-ID 21–40) there was satisfactory interrater agreement for all dosage forms except for the insulin pen. As in the first section for eye-drops, rating rules for the insulin pen were adapted (unscrew the green cap: sever difficulties, injection: not enough back pressure so the pen moves in hand, or several tries to press down: moderate difficulties) (see Supplementary Table S2) and training was repeated. JG and TD re-evaluated the insulin pen in this section again taking the new rating rules into account and achieved satisfactory interrater agreement.

In all further sections (patient-ID 41–60, 61–80 and 81–100) there was satisfactory interrater agreement for all dosage forms.

The intrarater agreement as determined by rating the self-administration performance of one patient at three different points of time was excellent for both JG (1.00 [0.99–1.00]) and TD (0.97 [0.91–0.99]).

Table 4 (see also Supplementary Tables S3–S4) shows the agreement of JG and TD to the reference standard for one patient in each section. In all cases there was satisfactory agreement. In summary we could show satisfactory agreement between the two raters (interrater reliability), between each rater across different points of time (intrarater reliability) and between each rater and the reference standard (accuracy).

**Table 4:** Dosage form-specific interrater agreement between JG/reference standard and TD/reference standard.

	Tablets	Eye-drops	Oral drops	Insulin pen	Patch
Rater JG versus reference standard	0.67 [−2.20–0.97]	1.00 [1.00–1.00]	1.00 [1.00–1.00]	0.80 [−0.97–0.98]	0.84 [−0.56–0.98]
Rater TD versus reference standard	0.52 [−3.62–0.95]	1.00 [1.00–1.00]	1.00 [1.00–1.00]	0.77 [−1.19–0.98]	0.92 [0.22–0.99]

Besides, the raters also evaluated the video quality of each recording (good, limited, or not usable). In total, 79% of the video recordings were rated to have good quality, 17% to have limited quality, and 4% as being not usable.

## Discussion

We report on the development of an evaluation procedure to objectively, reliably, and validly assess video recorded medication management performance including five different dosage forms in 67 older patients in the ABLYMED study. The pilot phase, during which 19 raters (of which two determined the reference standard by consensus) evaluated the video recorded medication management performance of three patients, confirmed the accuracy and precision of the standardized assessment form and rating rules. For the four dosage forms tablets, eye-drops, oral-drops and insulin pen, interrater agreement was satisfactory with an ICC between 0.54 and 1.00. Only the rating of the patches showed poor agreement for JG/reference standard, TD/median R1-R15 and JG/TD and led to the inclusion of an additional rating rule. In the rating phase, during which two raters evaluated the video recorded medication management performance of all 67 patients, interrater agreement (precision) was satisfactory with an ICC between 0.67 and 0.99, implying a range between moderate to excellent precision. In section 1 and 2 (patient-ID 1–20 and 21–40) additional rating rules for eye-drops and the insulin pen became necessary to reach satisfactory agreement. The agreement of the two raters with the reference standard (accuracy) at the end of the rating phase was satisfactory as well (ICC between 0.52–1.00). Furthermore, intrarater agreement over time at t0, t1 (after 2 weeks) and t2 (after 4 weeks) for JG (ICC = 1.00) and TD (ICC = 0.97) was excellent. To conclude, our results suggest that the evaluation procedure of the video recorded medication management performance for different dosage forms in older patients is valid and reproducible.

Insufficient medication management performance leads to impairments in the ability to engage in self-care and live independently (9). Thus, preserved medication performance skills are important for patients’ quality of life. Besides, they are essential for a safe drug therapy. Older patients are at a higher risk for patient medication errors such as incorrect dosage, forgetting, mixing up medications, incorrect handling of inhalers or inappropriately storing drugs (19). The video recordings help to uncover patient’s individual handling errors which influence medication management performance.

Prior studies using video recordings to measure patients’ medication management performance are scarce. Park et al. used video recordings to examine self-instillation of artificial tears in 78 patients with glaucoma or ocular hypertension. Three raters (medical students) evaluated the videos on the three criteria efficacy (whether an eye drop was instilled on the ocular surface), safety (whether the tip of the medication bottle made contact with the ocular surface or eyelids), and efficiency (number of eye drops expressed from the bottle). Park et al. reported good interrater reproducibility with a mean kappa level 0.64 for efficacy, 0.73 for safety, and 0.62 for efficiency. Measuring medication management performance using the criteria efficacy, safety, and efficiency is less detailed than our evaluation of each administration step. Furthermore, this study focused on eye-drops. In addition, our study comprised all common dosage forms to reach a comprehensive view of patients’ medication management performance (14). Of note, there are some prior studies about assessment tools measuring medication management performance. One tool is called Drug Regimen Unassisted Grading Scale (DRUGS) which was developed based on 59 outpatients at the age of 70 years and older. These patients had to perform the four tasks identification, access, dosage, and timing with their own medication. The performance was evaluated by the DRUGS tool score ranging from 0% (when a patient can perform none of the tasks for none of their own medication) to 100% (when a patient can perform all four tasks for every drug of the own medication). The authors reported a statistically significant association between performance in the tasks of access, dosage, timing, and the Mini-Mental State Exam (MMSE) score (20). Another tool is called Medication Management Instrument for Deficiencies in the Elderly (MedMaIDE) which is based on a study with 50 patients at the age of 65 years and older, living in the community and being self-medicating. Participants had to answer 20 items about knowledge, how to take their medication, and procurement. Besides, validity was assessed by comparing the MedMaIDE score to pill count adherence. MedMaIDE is a valid and reliable instrument to assess medication management performance in older adults, but relies exclusively on patients’ responses without external validation of handling skills (21). The assessment tool Medication Management Ability Assessment (MMAA) was developed based on 104 patients older than 45 years with schizophrenia and 33 normal comparison subjects, who had to perform a role-play task that simulated a fictious medication regime which was of similar complexity than those of older people. The total number of pills over that prescribed, total number of pills under that prescribed, and total number of correct responses were noted. Furthermore, adherence was measured by pharmacy claims data and cognitive status by MMSE. Patients made significantly more errors in the MMAA compared with controls and performance was significantly associated with prescription refill records and cognitive performance suggesting that adherence may improve with improving cognitive functions (22). The tools MMAA and DRUGS show a high correlation (Pearson correlation coefficient of 0.56) with each other in older individuals living in the community. Furthermore, the results of both tools correlated positively with cognitive function measured by MMSE (23).

Of note, contrary to our study, all the tools mentioned above only investigated single dosage forms but not all forms available. Furthermore, none of these instruments applied an objective, reliable, and ecologically valid quantitative assessment of medication management performance based on behavioral observations in standardized test settings as performed in the ABLYMED study. The methods applied in these studies to assess validity relied on adherence and cognition (16). In the ABLYMED study we assess adherence by Medication Adherence Report Scale (MARS), a tool already validated and widely used in clinical routine, and cognitive functions by six-item screener, Timed Test of Money Counting, Trail Making Test for older subjects (ZVT-G) and Clock-drawing test (8). Although prevention of patient’s medication errors is not the main objective of our study, previous studies showed, that improving information about medication use reduces medication errors (24). Besides, patients sometimes revealed their home-grown strategies to deal with the daily medication. For example, patients reported to use scissors or knifes to open packaging or to divide tablets. To reduce patients’ medication errors, methods like verbal instructions, tear-off calendars, apps and motivational interviews were examined so far (19). Pharmaceutical counseling on correct medication administration is not well established in all dosage forms. It is only common with inhalers or insulin pens (25, 26). Maybe pharmaceutical counseling on correct medication administration in all dosage forms can preserve medication performance skills. Furthermore, findings from the video recordings can indicate that a patient requires a different dosage form: When for example, a patient is not able to open a bottle of oral drops but is able to take tablets without any problems, the dosage form of tablets would be preferable if available. Of note, the ARMIN-Project (Arzneimittelinitiative Sachsen Thüringen) shows the importance of the interprofessional medication management program between community pharmacists and general practitioners to improve medication safety and effectiveness (27). The combination of individual counseling on correct medication administration and prescription of adequate medication preparations may reduce medication errors and preserve patients’ independence.

Our study considers a population that excludes patients with cognitive impairments in order to create a tool, that is valid in persons who usually handle their medication by themselves. These independent patients are common in primary care practices. They may be at risk for medication-related problems, although the risk is less obvious in this group of patients. That is why our research has high relevance in an aging society.

Nevertheless, there are some limitations of our study to be considered. We determined a reference standard for the videos of the pilot phase and for video recordings of five patients in the rating phase by two raters (AL, HF). We used the reference standard as a gold standard to measure accuracy. This is the result of the best available method (28) as no other accepted gold standard is available. The MedMaIDE could be used as an established reference with demonstrated validity and reliability. However, the usefulness of this instrument for our purposes is limited because of some missing dosage forms (only tablets, patches, and insulin pen) and because the observations are done by one person only (21). Other instruments such as Medication Management Evaluation Instrument (MMEI), Medication Management Test (MMT) and Medication Assessment Instruments (MAI) are less suitable due to limitations in validity or the investigated population (9). Nevertheless, other instruments like MMAA and Medication Management Evaluation Instrument (MMEI)were validated by using at least one related construct like cognitive function (Cognitive Capacity Screening Exam, neuropsychological test battery) or medication adherence (self-reported, pill counting or pharmacy claims data) (9). Because there is no existing instrument to objectively evaluate medication management performance of different dosage forms, in future work within the ABLYMED study, we are going to use different external validation steps (association with grooved pegboard performance as an indicator of manual dexterity and complex visual-motor coordination, with Six-Item-Screener as an indicator of cognitive function, with self-reported adherence and with MedMaIDE). Another limitation concerns the non-standardized video recording setting. As explained, we filmed the patients in their rooms with a smartphone camera. No further equipment or technical support was used to minimize intrusiveness and increase the ecological validity of the test setting. This came along with some limitations in video quality. In particular, there were some late onsets of the video recordings and suboptimal image sections. Late onset of the videos occurred when patients had no difficulties at all and managed the first administration steps quickly. We considered the presence of late onsets in our general rating rules, according to which missing steps are scored as no difficulties (see Supplementary Table S2). In total, 79% of the video recordings were rated to have good quality, 17% to have limited quality, and 4% as being not usable. Thus, we would argue that any negative impact of our non-standardized recording procedure is rather limited and outweighed by the ease of our procedure’s integration into the geriatric hospital setting. Furthermore, only two raters evaluated all videos. Because of the high workload the limitation on two raters is ecologically valid and realistic. Unexpectedly, some problems with the different dosage forms of medications occurred. First, in some videos the bottle of the oral drops was blocked. Therefore, executing the task was complicated for the participants. This occurred in ten cases. Therefore, we decided to implement this into the rating rules: the raters should evaluate whether patients took action (e.g., shake the bottle, turn the bottle, see Supplementary Table S2). However, blocked bottles of oral drops also exist in real-life settings with non-placebo drugs, increasing the ecological validity of our task. Despite previous testing we could not avoid this. Second, we had to change our patches from a product with subdivided protective liner to a product with continuous protective liner. The reason was a stop in production during data collection. Critical steps of patch application are opening the packaging and removing the protective foil (29). While the packaging and the protective liner differed between the two products and may have biased the absolute ratings, the inter- and intrarater agreement was not affected negatively. Of note, the comparison of different patches and their handling may also be an exciting topic for further research as it is already performed for different inhalers (27, 30). Regarding the different dosage forms of the medications, some aspects need to be discussed. In our study we only assessed opening a one-dose ophtiole dispenser of eye-drops. Due to data privacy, we were not allowed to film the eye-drop instillation. A study in patients with glaucoma showed, that especially placing the drops in both eyes and maintaining the bottle’s sterility during application are often improper (31). Thus, our performance task in the self-administration of eye-drops may have been too easy and may not cover critical steps of eye drop administration. Finally, we did not access inhaler use in our assessment battery, because this topic is well investigated (27, 32). Additionally, the number of patients in need of inhalers was too low to achieve reliable results.

## Conclusion

Using a typical older, non-demented patient population with polypharmacy and independent medication management, the video recordings of medication administration performance and their systematic evaluation yielded important results for the ABLYMED study. The satisfactory interrater and intrarater agreement of the ratings showed a valid and reproducible evaluation procedure of the video recordings. The rating results represent patients’ objective ability to self-administer medication. Due to the existing gap between patients’ self-reported ability to self-administer medication and their observable skills, reliable and valid measurement of actual medication management performance, as presented, is essential. One further step is the identification of factors influencing the ability to self-administer medication. This topic will also be analyzed in the ABLYMED study. Our results will be applicable to patients in a comparable health condition, that means patients without cognitive impairment and without any need of daily care. Such patients can be identified easily since they usually visit their family doctor independently and without help. Our method of constructing the assessment form to evaluate the performance of patients while self-administering different dosage forms of medication can function as a guide also for other patient populations since the assessment form can be adapted to their individual characteristics and problems. Factors influencing the ability to self-administer medication should be included in geriatric assessment to avoid medication errors and secure patients independent living.

## Data availability statement

The original contributions presented in the study are included in the article/Supplementary material, further inquiries can be directed to the corresponding author/s.

## Ethics statement

The studies involving human participants were reviewed and approved by Heinrich Heine Universität Düsseldorf. The patients/participants provided their written informed consent to participate in this study. Written informed consent was obtained from the individual(s) for the publication of any potentially identifiable images or data included in this article.

## Author contributions

AL, HF, SW, and JG developed the research question and the rating procedure. AL and HF collected patient data and determined the reference standard. JG and TD analyzed video recordings. JG performed statistical analyses of data together with AL. AL, JG, and HF drafted the manuscript. TD, RL, SW, and DH critically revised the manuscript. AL, RL, SW, TD, DH, HF, and JG agreed both to be personally accountable for their own contributions and to ensure that questions related to the accuracy or integrity of any part of the work, even ones in which they were not personally involved, are appropriately investigated, resolved, and the resolution documented in the literature. All authors contributed to the article and approved the submitted version.

## Funding

This study received funding from the Paul-Kuth Foundation, Wuppertal, Germany. The funder had no role in the design of the study and collection, management, analysis, and interpretation of data, writing the manuscript and decision to submit the manuscript for publication.

## Conflict of interest

The authors declare that the research was conducted in the absence of any commercial or financial relationships that could be construed as a potential conflict of interest.

The reviewer BB declared a past co-authorship with the author(s) TD, DH to the handling editor.

## Publisher’s note

All claims expressed in this article are solely those of the authors and do not necessarily represent those of their affiliated organizations, or those of the publisher, the editors and the reviewers. Any product that may be evaluated in this article, or claim that may be made by its manufacturer, is not guaranteed or endorsed by the publisher.

## Supplementary material

SUPPLEMENTARY VIDEOS S1-S23
https://figshare.com/articles/media/Video_S1-S23_mov/21383736
