# Supplementary material for: Developing a novel tool to assess the ability to self-administer medication – A systematic evaluation of patients’ video recordings in the ABLYMED study
Source: Front Med (Lausanne). 2023 Feb 16;10:1040528. doi: 10.3389/fmed.2023.1040528 (PMC9978218; doi:10.3389/fmed.2023.1040528)
Supplement: SUPPLEMENTARY VIDEOS S1-S23 — https://figshare.com/articles/media/Video_S1-S23_mov/21383736 [file Data_Sheet_1.PDF]

Supplementary Material

Table S1. Assessment form for the video ratings

**Tablets**			
White tablet removal from the blister pack	no difficulties	1	
	mild difficulties	2	
	moderate difficulties	3	
	severe difficulties	4	
	not possible	5	
Blue tablet removal from the tablet tube	no difficulties	1	
	mild difficulties	2	
	moderate difficulties	3	
	severe difficulties	4	
	not possible	5	
Cutting the blue tablet	no difficulties	1	
	mild difficulties	2	
	moderate difficulties	3	
	severe difficulties	4	
	not possible	5	
	correct	1	

Correctly filling the pill organizer	incorrect	5	
**sum score**			
**Eye-drops**			
Open the one-dose ophtiole dispenser	no difficulties	1	
	mild difficulties	2	
	moderate difficulties	3	
	severe difficulties	4	
	not possible	5	
**sum score**			
**Oral drops**			
Open the child-resistant dropper bottle	no difficulties	1	
	mild difficulties	2	
	moderate difficulties	3	
	severe difficulties	4	
	not possible	5	
Targeting at the teaspoon	no difficulties	1	
	mild difficulties	2	
	moderate difficulties	3	
	severe difficulties	4	

	not possible	5	
Correct number of drops(n=10) on the teaspoon	correct	1	
	incorrect	5	
**sum score**			
**Insulin pen**			
Remove the transparent cap of the pen	no difficulties	1	
	mild difficulties	2	
	moderate difficulties	3	
	severe difficulties	4	
	not possible	5	
Remove the green cap of the needle	no difficulties	1	
	mild difficulties	2	
	moderate difficulties	3	
	severe difficulties	4	
	not possible	5	
Dialing in the right dose (12 units)	no difficulties	1	
	mild difficulties	2	
	moderate difficulties	3	
	severe difficulties	4	
	not possible	5	

Injection into a ball	no difficulties	1	
	mild difficulties	2	
	moderate difficulties	3	
	severe difficulties	4	
	not possible	5	
**sum score**			
**Patch**			
Unpack the patch	no difficulties	1	
	mild difficulties	2	
	moderate difficulties	3	
	severe difficulties	4	
	not possible	5	
Peeling off the protective liner	no difficulties	1	
	mild difficulties	2	
	moderate difficulties	3	
	severe difficulties	4	
	not possible	5	
Apply it onto the skin	no difficulties	1	
	mild difficulties	2	
	moderate difficulties	3	

	severe difficulties	4	
	not possible	5	
**sum score**			

Table S2. Rating rules for the video ratings

Tablets

- Slider does not necessarily have to be closed before splitting the tablet, even if not closed this implies no difficulties.
- Any difficulties with the drug packaging should be included in the evaluation of the white tablet/blue tablet removal (e.g., blister return into the wrong packaging, difficulties in closing the packaging)
- If tablets are placed on top of the slider, it should be included in the evaluation of the white tablet removal (one tablet on the slider: mild difficulties, two tablets on the slider: moderate difficulties, three tablets on the slider: severe difficulties)
- Cutting the tablet: Quality of division is not evaluated, multiple tries of pushing down should be evaluated with moderate or severe difficulties

Eye-drops

- Evaluation is based on the number of grasping movements (one to two: no difficulties, three to four: mild difficulties, five to six: moderate difficulties, more than six: severe difficulties)

Oral drops

- If the evaluation of correct counting is no possible, the rating is replaced by the counting of AL during the video recording.
- In some video recordings the bottle is blocked. In this case for the step “targeting at the teaspoon” the raters should evaluate whether patients take action (e.g. shake the bottle, turn the bottle), in some cases a video recording follows just counting the drops after the experimenter has checked the bottle.

Insulin pen

- Removal of the green cap does not work without support: moderate difficulties
- Unscrewing the green cap: severe difficulties because the needle is missing
- Dialing in the right dose does not work without support but afterwards fluid fulfillment of the step: moderate difficulties
- If the plunger is not pushing all the way through during injection: severe difficulties
- Injection: Not enough back pressure so the pen moves in hand, or several tries to press down: moderate difficulties

Patch

- Patch 1: It is possible to peel off both protective liners together, if there is little contact to the sticky area of the patch this implies no difficulties.
- Patch 2: Time until removing the first protective liner: Up to 15 sec: no difficulties, 16-30 sec.: mild difficulties, 31-45 sec.: moderate difficulties, 46-60 sec. severe difficulties, second protective liner is not removed: moderate difficulties
- It is not relevant where the patient sticks the patch (e.g., arm, back of the hand).

General rules

- Video starts too late: Evaluation of the missing steps as no difficulties
- Video aborts: Step is not possible, all further steps also not possible
- Video interruption: step is not possible, video continues after practical assistance
- Examinator's hands seen on video indicates assistance and thus difficulties (once per step: moderate difficulties, twice per step: severe difficulties)

Table S3. Dosage form-specific mean sum scores in the pilot phase

	**tablets**	**eye-drops**	**oral drops**	**insulin pen**	**patch**
**median of R1-R15 vs reference standard**	7.3 vs 7.3	1.7 vs 1.7	5.3 vs 6.0	6.3 vs 7.0	3.7 vs 3.7
**rater JG vs reference standard**	6.0 vs 7.3	2.0 vs 1.7	6.3 vs 6.0	7.0 vs 7.0	3.7 vs 3.7
**rater TD vs reference standard**	6.0 vs 7.3	2.3 vs 1.7	5.3 vs 6.0	7.7 vs 7.0	3.0 vs 3.7
**R1-R15**	-	-	-	-	-
**median of R1-R15 vs rater JG**	7.3 vs 6.0	1.7 vs 2.0	5.3 vs 6.3	6.3 vs 7.0	3.7 vs 3.7
**median of R1-R15 vs rater TD**	7.3 vs 6.0	1.7 vs 2.3	5.3 vs 5.3	6.3 vs 7.7	3.7 vs 3.0
**rater JG vs rater TD**	6.0 vs 6.0	2.0 vs 2.3	6.3 vs 5.3	7.0 vs 7.7	3.7 vs 3.0

Table S4. Dosage form-specific mean sum scores in the rating phase

**Dosage form-specific interrater agreement for patient 1-20**					
	tablets	eye-drops	oral drops	insulin pen	patch
rater JG vs TD	7.9 vs 6.0	1.6 vs 1.1	5.3 vs 3.7	8.1 vs 7.6	6.1 vs 5.3
**Dosage form-specific interrater agreement for patient 1-20 after second evaluation of eye-drops**					
	tablets	eye-drops	oral drops	insulin pen	patch
rater JG vs TD	7.9 vs 5.9	1.4 vs 1.8	5.3 vs 3.7	8.1 vs 7.6	6.1 vs 5.3
**Dosage form-specific interrater agreement for patient 21-40**					
	tablets	eye-drops	oral drops	insulin pen	patch
rater JG vs TD	6.9 vs 5.9	1.7 vs 1.8	4.9 vs 3.8	5.9 vs 4.1	4.6 vs 4.3
**Dosage form-specific interrater agreement for patient 21-40 after second evaluation of the pen**					
	tablets	eye-drops	oral drops	insulin pen	patch
rater JG vs TD	6.9 vs 5.9	1.7 vs 1.8	4.9 vs 3.8	5.9 vs 5.4	4.6 vs 4.3
**Dosage form-specific interrater agreement for patient 41-60**					
	tablets	eye-drops	oral drops	insulin pen	patch
rater JG vs TD	7.5 vs 7.3	1.4 vs 1.7	5.6 vs 4.7	7.3 vs 7.2	6.7 vs 6.4
**Dosage form-specific interrater agreement for patient 61-80**					
	tablets	eye-drops	oral drops	insulin pen	patch
rater JG vs TD	7.7 vs 8.2	1.3 vs 1.9	4.7 vs 3.7	6.2 vs 6.5	5.8 vs 6.9
**Dosage form-specific interrater agreement for patient 81-100**					
	tablets	eye-drops	oral drops	insulin pen	patch
rater JG vs TD	5.9 vs 8.0	1.2 vs 1.4	4.8 vs 4.7	7.6 vs 7.4	5.6 vs 5.9
**Dosage from-specific mean sum scores JG vs reference standard and TD vs reference standard for patient 1-100**					
	**tablets**	**eye-drops**	**oral drops**	**insulin pen**	**patch**
**rater JG vs reference standard**	6.0 vs 5.0	1.0 vs 1.0	3.0 vs 3.0	4.6 vs 5.8	4.4 vs 4.2
**rater TD vs reference standard**	6.2 vs 5.0	1.0 vs 1.0	3.0 vs 3.0	6.2 vs 5.8	4.6 vs 4.2
